# The Traf2 and NcK interacting kinase inhibitor NCB-0846 suppresses seizure activity involving the decrease of GRIA1

**DOI:** 10.1016/j.gendis.2023.03.036

**Published:** 2023-06-24

**Authors:** Min Wang, Yixue Gu, Qiubo Li, Bangzhe Feng, Xinke Lv, Hao Zhang, Qingxia Kong, Zhifang Dong, Xin Tian, Yanke Zhang

**Affiliations:** a Department of Neurology, Affiliated Hospital of Jining Medical University, Jining, Shandong 272000, China; b Department of Neurology, The First Affiliated Hospital of Chongqing Medical University, Chongqing Key Laboratory of Neurology, Chongqing 400016, China; c Department of Pediatrics, Affiliated Hospital of Jining Medical University, Jining, Shandong 272000, China; d Department of Neurosurgery, Affiliated Hospital of Jining Medical University, Jining, Shandong 272000, China; e Pediatric Research Institute, Ministry of Education Key Laboratory of Child Development and Disorders, National Clinical Research Center for Child Health and Disorders, Chongqing Key Laboratory of Translational Medical Research in Cognitive Development and Learning and Memory Disorders, Children's Hospital of Chongqing Medical University, Chongqing 400014, China; f Center for Excellence in Brain Science and Intelligence Technology, Chinese Academy of Sciences, Shanghai 200031, China

**Keywords:** Epilepsy, NCB-0846, Rat, TLE, TNIK, Traf2-and NcK-interacting kinase

## Abstract

Epilepsy, one of the most common neurological disorders, is characterized by spontaneous recurrent seizures. Temporal lobe epilepsy (TLE) is one of the most common medically intractable seizure disorders. Traf2-and NcK-interacting kinase (TNIK) has recently attracted attention as a critical modulation target of many neurological and psychiatric disorders, but its role in epilepsy remains unclear. In this study, we hypothesized the involvement of TNIK in epilepsy and investigated TNIK expression in patients with intractable TLE and in a pilocarpine-induced rat model of epilepsy by western blotting, immunofluorescence, and immunohistochemistry. A pentylenetetrazole (PTZ)-induced epilepsy rat model was used to determine the effect of the TNIK inhibitor NCB-0846 on behavioral manifestations of epilepsy. Coimmunoprecipitation (Co-IP)/mass spectrometry (MS) was used to identify the potential mechanism. Through Co-IP, we detected and confirmed the main potential TNIK interactors. Subcellular fractionation was used to establish the effect of NCB-0846 on the expression of the main interactors in postsynaptic density (PSD) fractions. We found that TNIK was primarily located in neurons and decreased significantly in epilepsy model rats and TLE patients compared with controls. NCB-0846 delayed kindling progression and decreased seizure severity. Co-IP/MS identified 63 candidate TNIK interactors in rat hippocampi, notably CaMKII. Co-IP showed that TNIK might correlate with endogenous GRIA1, SYN2, PSD-95, CaMKIV, GABRG1, and GABRG2. In addition, the significant decrease in GRIA1 in hippocampal total lysate and PSDs after NCB-0846 treatment might help modify the progression of PTZ kindling. Our results suggest that TNIK contributes to epileptic pathology and is a potential antiepileptic drug target.

## Introduction

Epilepsy, which is characterized by spontaneous recurrent seizures, is one of the most prevalent neurological disorders. Approximately 1% of the global population is affected by epilepsy; specifically, temporal lobe epilepsy (TLE) is the most common type of epilepsy in adults. An estimated one-third of patients are resistant to current medication therapy.^1^^,^^2^ Furthermore, patients with seizures feel stigmatized and report reduced quality of life. Therefore, it is of great importance to find new drug targets to develop novel therapeutic strategies for epilepsy, especially refractory epilepsy. However, the development of treatments for epilepsy is hampered by a lack of mechanistic knowledge about this common neuropsychiatric condition. The hippocampus is one of the main epileptogenic regions identified in animal model studies, and it is also one of the most common seizure foci indicated by neuropathological findings in TLE patients.^3^ Dendritic spines in excitatory afferents are critical for the formation of functional neural circuits. Excitation/inhibition (E/I) imbalance in the neural circuitry is considered one of the most important pathogenetic mechanisms of epilepsy.^4^ Increasing evidence shows that aberrant synaptic morphology is related to several neurological disorders, including epilepsy, intellectual disability, Alzheimer's disease, and schizophrenia.^5^^,^^6^ However, the pathogenesis and mechanism of epilepsy are still unclear and need to be further characterized.

Traf2-and NcK-interacting kinase (TNIK) was first identified by two-hybrid screening in 1999 as a novel member of the germinal center kinase (GCK) family. The GCK family, which interacts with tumor necrosis factor (TNF)-receptor-associated factor 2 (TRAF2) and NCK adaptor protein 1 (NCK1), is a subgroup of the STE20 kinase family.^7^ TNIK is highly and widely expressed in the mammalian brain, including the cortex and hippocampus, especially in dentate gyrus granule cells,^8^^,^^9^ but its expression is weaker in the midbrain, pons, and medulla.^10^ TNIK, which has both scaffolding domains and enzymatic activity, has been implicated in cell proliferation, cytoskeleton organization, neuronal dendrite extension, and glutamate receptor regulation *in vitro*.^7^^,^^11^^,^^12^ Furthermore, previous studies showed that TNIK might be one of the components of the postsynaptic density (PSD) and synaptosomal fractions.^13^, ^14^, ^15^ Knockdown of TNIK resulted in a significant reduction in the density of dendritic spines.^15^ In addition, TNIK has been shown to be involved in postsynaptic signaling,^8^^,^^9^^,^^15^ cytoskeleton organization, and neuronal dendrite extension. In addition, TNIK has been reported to bind protein complexes in the synapse, linking it to NMDA-type glutamate receptors (NMDARs).^8^^,^^9^ In primary neuron cultures, TNIK also interacts with the psychiatric risk-associated protein DISC1 (disrupted in schizophrenia 1)^16^ and regulates postsynaptic density protein 95 (PSD-95).^9^ In addition, TNIK has been shown to play a significant role in promoting surface expression of the AMPA-type glutamate receptor (AMPAR) subunit GRIA1 in primary neurons.^9^^,^^15^ In addition, TNIK knockout mice displayed hyperlocomotion.^8^ Although AMPAR- and NMDAR-mediated synaptic currents, spontaneous inhibitory postsynaptic currents, miniature inhibitory postsynaptic currents (mIPSCs), long-term potentiation, and long-term depression were normal in the hippocampal CA1 of TNIK knockout mice, a previous study observed a significant increase in paired-pulse facilitation and a significant decrease in the frequency of AMPA receptor-mediated miniature excitatory postsynaptic currents (mEPSCs) in TNIK^−/−^ mice.^8^ The use of a synthesized TNIK-inhibiting peptide down-regulates cell-surface GRIA1 levels and AMPAR-mediated currents in rat hippocampal cultures.^9^ Moreover, using genetic and bioinformatic methods, many recent reports have proposed TNIK as an anticancer target molecule^17^ in many types of cancers, such as colorectal cancer,^18^, ^19^, ^20^ hepatocellular carcinoma,^21^ lung cancer,^22^ synovial sarcoma,^23^ prostate cancer,^24^ and breast cancer.^25^ TNIK has been shown to have potential importance in schizophrenia,^9^^,^^26^, ^27^, ^28^ attention-deficit/hyperactivity disorder (ADHD),^29^ and bipolar disorder.^30^ TNIK is also involved in other pathological processes or diseases, such as neurodevelopmental disorders,^31^ intellectual disability (ID),^32^ and neuropathic allodynia,^33^ and it has been shown to play a role in cognitive function.^8^

Excitatory synaptic transmission, which is mediated by activation of AMPARs and NMDARs, is associated with epilepsy. Based on previous genetic studies and the significance of TNIK in the function of schizophrenia, ID, glutamate receptors, and spine morphology, we hypothesized that TNIK may participate in the pathology of TLE and may be a potential therapeutic target for epilepsy. To test our hypothesis, we evaluated the expression pattern of TNIK in the brains of TLE patients and epileptic rat models and tested the effect of the TNIK inhibitor NCB-0846 in a pentylenetetrazole (PTZ)-induced rat model of epilepsy. We found that TNIK was down-regulated in epilepsy, and the inhibition of TNIK using NCB-0846 alleviated seizure activity. Furthermore, Co-IP/MS together with western blotting showed that TNIK interacted with CaMKII, SYN2, CaMKIV, GRIA1, GABRG1, GABRG2, and PSD-95 but not with GRIA2, GRIN1, GRIN2A, GRIN2B, GABRA1, GABRA3, GABRA4, GABRB1, or vGlut1. GRIA1 redistributed out of the PSD after NCB-0846 treatment, which might contribute to the decrease in seizure activity. In addition, NCB-0846 treatment increased the expression of CaMKIV but not CaMKII. A decrease in GABRG2 but not GABRG1 might also be involved in the contribution of TNIK to epilepsy. Thus, our findings provide valuable information about the mechanisms of epilepsy.

## Materials and methods

### Patient selection

All human brain tissue specimens were obtained as described in our previous study.^34^, ^35^, ^36^ Twenty cortical tissue samples from TLE patients and 10 cortical tissue samples from control subjects were included in the present study. The inclusion criteria for TLE patients were typical epilepsy symptoms, a detailed medical history, distinct electroencephalogram (EEG) findings, a neurological examination, neuroimaging, and persistence of seizures despite more than 2 years of medical therapy with 3 or more types of antiepileptic drugs (AEDs) at their respective effective blood concentrations (defined as refractory epilepsy). Patients' epileptic lesions were localized by intraoperative electrocorticography. Cortical samples from TLE patients were obtained only for treatment purposes. Control subjects were patients treated for increased intracranial pressure due to head trauma requiring surgery but without a history of epilepsy, exposure to AEDs, or other neurological diseases. The detailed clinical characteristics of the TLE patients and control subjects are shown in Table 1, Table 2, respectively. All enrolled patients or their family members voluntarily joined this study and signed informed consent forms. All protocols involving humans were approved by the Committee on Human Research at Jining Medical University, as well as the Declaration of Helsinki from the World Medical Association.

**Table 1:** Clinical characteristics of TLE patients.

Patient number	Course (y)	Sex (M/F)	Age (y)	AEDs before surgery	Pathology	Side of resected temporal lobe
1	12	M	20	CBZ, PHT, TPM	NL	L
2	25	M	45	LTG, PB, TPM, PHT	NL	L
3	8	M	12	GBP, CBZ, PTH, LEV	NL	L
4	15	M	17	VPA, CBZ, TPM, LTG	NL	L
5	12	M	20	PHT, LTG, VPA	NL, gliosis	L
6	11	M	23	PHT, CBZ, LEV, VPA, PB	NL	R
7	11	M	22	CBZ, VPA, LTG, LTG	Gliosis	L
8	30	M	35	VPA, CBZ, PB, PHT	NL	L
9	16	M	35	PB, VPA, TPM, LEV	NL	L
10	10	M	55	PB, TPM, LTG	NL	R
11	16	F	36	PB, LEV, CBZ, PHT	Gliosis	L
12	10	F	50	CBZ, PB, TPM	NL	R
13	12	F	35	CBZ, TPM, PHT	NL, gliosis	R
14	12	F	16	CBZ, TPM, LEV	NL	L
15	10	F	13	OXC, PB, CBZ, PHT	Gliosis	R
16	14	F	40	VPA, CBZ, PHT, LEV	NL	R
17	10	F	33	PB, CBZ, PHT	Gliosis	L
18	12	F	15	PHT, PB, VPA, LEV	NL, gliosis	L
19	16	F	45	LEV, CBZ, PHT	Gliosis	R
20	7	F	23	OXC, PB, PHT	Gliosis	R

y year, AEDs antiepileptic drugs, CBZ carbamazepine, VPA valproic acid, PB phenobarbital, PHT phenytoin, GBP gabapentin, TPM Topamax, LTG lamotrigine, OXC oxcarbazepine, LEV levetiracetam, L left, R right, NL neuron loss, M male, F female.

**Table 2:** Clinical characteristics of control patients with head trauma.

Patient number	Sex (M/F)	Age (y)	Pathology	Side of resected temporal lobe
1	M	35	RN	L
2	M	23	RN	R
3	M	34	RN	L
4	M	15	RN	L
5	M	32	RN	L
6	F	54	RN	R
7	F	44	RN	R
8	F	11	RN	R
9	F	21	RN	R
10	F	30	RN	L

L left, R right, RN relative normal.

### Epilepsy rat model construction

Healthy adult male Sprague‒Dawley (SD) rats purchased from Pengyue (Jinan, Shandong, China) weighing 210–230 g were used in this study. The rats were housed in a temperature-controlled (22–24 °C) environment on a 12/12-h light/dark cycle and received water and food *ad libitum*. Rats were randomly divided among all groups as described below. All tests were conducted during the light phase. The Animal Ethics Committee of Jining Medical University approved all procedures, which were conducted in accordance with international standards. All efforts were made to minimize the number of experimental subjects used in this study and their suffering.

The pilocarpine-induced epilepsy model was established as described in our previous study,^37^ with some modifications. Pilocarpine hydrochloride (300 mg/kg, Sigma) was dissolved in bacteriostatic 0.9% NaCl and injected intraperitoneally into each rat. To antagonize the peripheral side effects of pilocarpine, atropine sulfate (1 mg/kg) was administered intraperitoneally 30 min before pilocarpine treatment. Status epilepticus (SE) was assessed by the presence of continuous and/or repetitive motor convulsions and confirmed by hippocampal local field potential (LFP) recording. Seizures were scored according to Racine's standard criteria (Racine, 1972). After 60 min of SE, seizures were terminated using diazepam (10 mg/kg, intraperitoneally). Healthy, nonpilocarpine-injected male SD rats of similar weight and age were used as the control group. Two months after SE, the epileptic rats were confirmed using LFP recording and sacrificed for Western blot analysis, immunofluorescence (IF), and immunohistochemistry (IHC).

The PTZ-kindled epilepsy model was established as described previously.^38^ Solvent or NCB-0846 was given 30 min before PTZ administration and continued for 14 days. PTZ (35 mg/kg, Sigma‒Aldrich Co., St. Louis, MO, USA) was administered to the rats intraperitoneally every other day. Then, the rats were monitored for at least 30 min to assess seizure activity by the Racine scale (Racine, 1972): stage I, immobility and staring; stage II, rigid posture; stage III, repetitive movements and head bobbing; stage IV, rearing and myoclonic twitching; and stage V, generalized tonic‒clonic seizures with falling. Rats with at least three consecutive seizures scored 4 or 5 were considered fully kindled. Latency time in the PTZ kindling model was defined as the last day with three consecutive seizures of scores 4 and 5.

### Surgical procedure

Intracerebroventricular cannulae for drug administration and electrodes for LFP recording were implanted as described in our previous paper.^37^ For surgical procedures, rats were first anesthetized using 5% isoflurane, and then 1% isoflurane was used to maintain stable anesthesia. During surgery, the respiratory rate was observed closely to monitor adequate anesthesia. Under anesthesia, rats were fixed in a stereotaxic apparatus (RWD Life Science, Co., Ltd, Shenzhen, China). The left lateral ventricle was targeted at coordinates of AP = 1.0 mm, ML = 1.5 mm on the left side, and DV = 3.5 mm from the dura. Cement was used to fix the accessories to the skull. Tests were conducted seven days after surgery and three days after environmental adaptation.

To confirm the success of SE construction and the pilocarpine-induced chronic epilepsy model, a microwire array was implanted into the right dorsal hippocampus (AP, ML, DV (mm): 3.6, 2.8, 3.6). Two reference screws were implanted in the skull. All accessories were fixed in the skull and used for LFP recording.

### Drugs

NCB-0846 (CAS No.: 1792999-26-8) was purchased from Selleck (Houston, TX, USA), dissolved in DMSO, and then diluted in bacteriostatic 0.9% NaCl. NCB-0846 (10 μM) or the solvent control was intracerebroventricularly injected into the cerebral ventricle via a cannula (0.5 μL/min, 10 μM, 5 μL).^24^^,^^25^

### LFP recording and analysis

Hippocampal LFP recordings were conducted to assess abnormal discharges. The signals were filtered (0.1–1000 Hz), amplified 1000 × , and digitized at 4 kHz using an OmniPlex® D neural data acquisition system (Plexon, Dallas, TX, USA). Spontaneous recurrent seizures were defined as a cluster of paroxysmal discharges with a frequency greater than 5 Hz and a high amplitude of spike activity more than 2 SDs from the baseline, lasting for 5 s or more.^34^^,^^37^

### Western blot and coimmunoprecipitation (Co-IP)

Samples from TLE patients and control subjects, as well as rat cortex and hippocampus in different groups, were homogenized in RIPA lysis buffer (Beyotime Institute of Biotechnology, China) and then centrifuged at 4 °C (14,000 g × 10 min). Hippocampal tissue for subcellular fraction collection was collected after NCB-0846 treatment for seven continuous days in naïve rats. The procedure for synaptosomal protein acquisition was conducted as described in our previous paper.^37^ In brief, the tissue was homogenized in ice-cold lysis buffer with protease inhibitors (Roche Applied Science) and phosphatase inhibitors (Sigma‒Aldrich) and then centrifuged at 100,000 g for 1 h at 4 °C. Pellets were resuspended in the same buffer containing 0.5% Triton X-100 and layered on sucrose (1 M). Then, the suspensions were centrifuged at 100,000 *g* for 1 h at 4 °C. Triton-insoluble material (highly enriched in PSD) sedimented through the sucrose layer and was resuspended in the same buffer containing 1% SDS.

The concentration of pure protein was determined using the bicinchoninic acid (BCA) protein assay (Beyotime Institute of Biotechnology). Equal quantities of prepared proteins were separated by SDS–polyacrylamide gel electrophoresis (SDS–PAGE; 5% spacer gel, 90 V; 10% separating gel, 120 V) and then transferred to a polyvinylidene fluoride (PVDF) membrane (300 mA). Next, the PVDF membrane was blocked with 5% skim milk and incubated with polyclonal mouse anti-TNIK (1:200, Santa), rabbit anti-GRIA1 (1:1000, Abcam), rabbit anti-SYN2 (1:500, Proteintech), rabbit anti-GRIA2 (1:500, Proteintech), mouse anti-GRIN1 (1:1000, synaptic systems), rabbit anti-GRIN2A (1:1000, Abcam), rabbit anti-GRIN2B (1:2000, Abcam), mouse anti-PSD-95 (1:1000, CST), rabbit anti-vGlut1 (1:500, Proteintech), rabbit anti-GABRB1 (1:500, Proteintech), rabbit anti-GABRA1 (1:500, Proteintech), rabbit anti-GABRA3 (1:500, Proteintech), rabbit anti-GABRA4 (1:500, Proteintech), rabbit anti-GABRG1 (1:500, Proteintech), rabbit anti-GABRG2 (1:500, Proteintech), rabbit anti-CaMKIV (1:1000, Abcam), and rabbit anti-CaMKII (1:1000, Abcam) diluted in 5% skim milk at 4 °C overnight. The membranes were washed with Tween-20 in Tris-buffered saline (TBST) and then incubated with horseradish peroxidase–conjugated goat anti-rabbit IgG antibody or horseradish peroxidase–conjugated goat anti-mouse IgG antibody (1:2000, ABclonal) for 2 h at 37 °C. A rabbit anti-GAPDH antibody (1:2000, ABclonal) was used as a loading control. Densitometry quantitation was measured using Quantity One 1-D Analysis Software (Bio-Rad Laboratories, Hercules, USA) as optical density (OD) values, and TNIK levels were normalized to GAPDH.

Co-IP assays were conducted as previously described.^38^ Rat hippocampal tissues were homogenized using IP buffer (Beyotime Institute of Biotechnology, China) containing protease inhibitors (Roche Applied Science) and phosphatase inhibitors (Sigma‒Aldrich) and were then prepared with mouse anti-TNIK (Santa Cruz Biotechnology) or a control consisting of normal mouse IgG (Millipore, USA) at 4 °C overnight. Then, they were incubated with protein G-agarose beads (Roche, Germany) at 4 °C for 3 h. The beads were then washed five times, collected, mixed with 2 × loading buffer, and heated at 95 °C for 5 min.

### Immunofluorescence (IF)

IF was conducted as previously described,^36^ with some modifications. The frozen sections of the samples from different groups were incubated in normal goat serum and then with a mixture containing a TNIK antibody (sc-377215, mouse polyclonal antibody, 1:100), a glial fibrillary acidic protein (GFAP) antibody (rabbit polyclonal antibody, 1:100, Proteintech), and a microtubule-associated protein 2 (MAP2) antibody (chicken polyclonal antibody, 1:1000, GeneTex) overnight at 4 °C. Sections were washed and incubated with a mixture of DyLight 488-conjugated goat anti-mouse IgG (1:1000, Abcam), DyLight 405-conjugated goat anti-rabbit IgG (1:1000, Abcam), and DyLight 555-conjugated goat anti-chicken IgG (1:1000, Abcam) in a darkroom for 120 min at 37 °C. After washing, the sections were mounted with 80% glycerol. Fluorescence was detected using laser scanning confocal microscopy (Leica Microsystems Heidelberg GmbH, Germany) on an Olympus IX 70 inverted microscope (Olympus, Japan) equipped with a Fluoview FVX confocal scan head.

### Immunohistochemistry (IHC)

IHC was conducted according to the manufacturer's protocol. Briefly, the sections were deparaffinized, processed for antigen recovery, blocked in goat serum (Wuhan Boster Biological Technology, Wuhan, China) and incubated in TNIK antibody (sc-377215, mouse polyclonal antibody, 1:50) overnight at 4 °C. The next day, the samples were incubated in goat anti-mouse secondary antibody for 30 min at 37 °C. Counterstaining was carried out with Harris's hematoxylin. For negative controls, the primary antibodies were replaced with PBS. Ten visual field images were randomly obtained from every section using an OLYMPUS PM20 automatic microscope (Olympus, Japan) and TCFY-2050 (Yuancheng Inc., China) pathology system.

### Co-IP coupled to mass spectrometry (Co-IP/MS)

Co-IP/MS was conducted as described in our previous paper with some modifications.^39^ Polyacrylamide gel was prepared in accordance with the standard protocol. In brief, purified protein samples prepared from Co-IP were separated by SDS‒PAGE and then stained with a Fast Silver Stain Kit (Beyotime, China). The desired pieces were cut from the SDS‒PAGE gel, and the stain was removed with 30% acetonitrile (ACN)/100 mM NH_4_HCO_3_. The proteins were reduced, alkylated, and digested by trypsin solution. The peptides were extracted three times with 60% ACN/0.1% trifluoroacetic acid (TFA). The extracts were pooled and dried completely with a vacuum centrifuge. LC‒MS/MS analysis was conducted by Applied Protein Technology (Shanghai, China) on a Q Exactive mass spectrometer (Thermo Fisher Scientific) that was coupled to an EASY-nLC (Thermo Fisher Scientific).

MS/MS spectra were searched using the Mascot engine (Matrix Science, London, UK; version 2.2) against the UniProt Rattus Norvegicus database (37195 sequences, downloaded on December 7, 2020). For protein identification, the following options were used: peptide mass tolerance = 20 ppm; fragment mass tolerance = 0.1 Da; enzyme = trypsin; missed cleavage = 2; fixed modification = carbamidomethyl I; filter by score ≥20; variable modification = oxidation (M).

### Bioinformatics analysis

Functional annotation and classification of all identified proteins were determined by using the Blast2GO program against the UniProt database. Pathway analyses were extracted using the search pathway tool of the Kyoto Encyclopedia of Genes and Genomes (KEGG) Mapper platform (http://www.genome.jp/kegg/mapper.html) and WebGestalt (http://www.webgestalt.org/). Potential interactions between those identified genes were displayed using GeneMANIA analysis (www.genemania.org).^40^

### Statistical analysis

The samples were analyzed in triplicate in each experiment. All data are presented as the mean ± SEM. Independent-samples Student's *t*-tests or the *χ*^2^ test were used to compare the differences between two groups using SPSS 19.0 software. Group differences in the mean seizure score during PTZ kindling were evaluated with repeated-measures ANOVA. Nonparametric tests were conducted when appropriate. Statistical significance was defined as *P* < 0.05.

## Results

### Clinical characteristics of human subjects

Twenty TLE patients (10 males and 10 females; mean age: 29.5 ± 2.92 years; age range: 12–55 years) with intractable epilepsy were included in this study. The average disease course was 13.4 ± 1.22 years (range: 7–30 years). All patients were diagnosed with epilepsy refractory to AEDs. Ten control subjects (5 males and 5 females; mean age: 29.9 ± 4.12 years; age range: 11–54 years) were included in this study. No significant differences in age or sex were found between the two groups (*P* > 0.05).

### Pilocarpine-induced rat model of epilepsy

**Figure 1:**
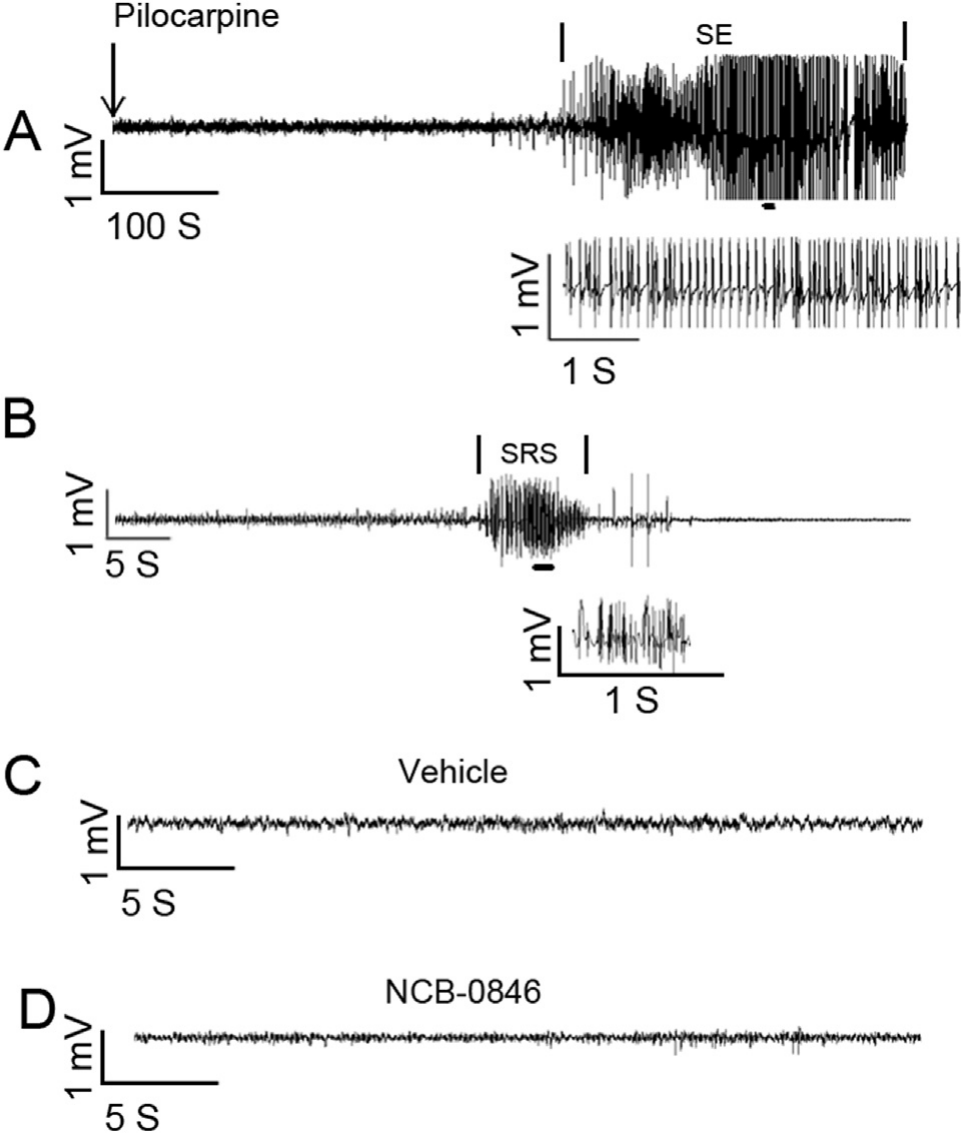
Typical LFP in this study. **(A)** Representative recordings of pilocarpine-induced status epilepticus in naïve rats. **(B)** Representative recordings of spontaneous paroxysmal discharges. **(C)** Representative figure of LFP under vehicle treatment. **(D)** Representative figure of LFP under NCB-0846 treatment (intracerebroventricularly, 10 μM, 5 μL).

After pilocarpine was administered, the rats gradually progressed to full electrographic SE, which was characterized by high-frequency and large-amplitude population spikes. In this study, 80% (16/20) of the rats entered SE with a single injection of pilocarpine (300 mg/kg). A typical electrographic recording of SE is shown in Figure 1A. Two rats died afterward, and the remaining rats were maintained for 2 months. Two months after SE, epileptic rats with typical spontaneous paroxysmal discharges were confirmed. A typical LFP is shown in Figure 1B. Vehicle or NCB-0846 treatment alone did not induce abnormal discharges (Fig. 1C, D).

### TNIK expression in the cortex of patients with TLE

**Figure 2:**
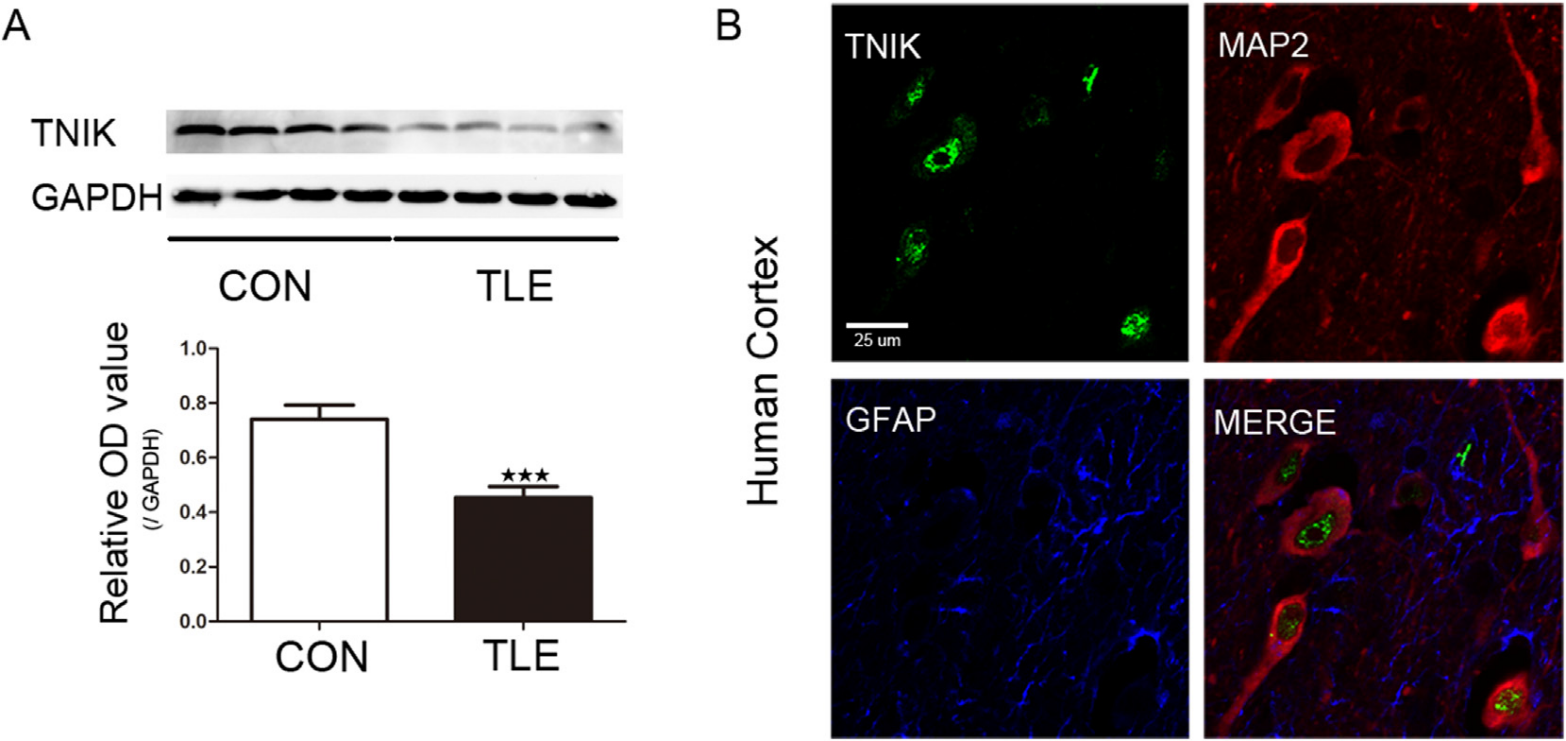
TNIK expression in the cortex of TLE patients. **(A)** TNIK expression was significantly decreased in the TLE group compared with the control group (*P* < 0.001). The relative OD ratio represents the OD ratio of TNIK relative to GAPDH. **(B)** Triple-label immunofluorescence demonstrated that TNIK (green) and GFAP (blue) were not co-expressed in astrocytes, but TNIK (green) and MAP2 (red) were co-expressed in cortical neurons of TLE patients (*n* = 5).

Western blotting and immunofluorescence were used to evaluate the expression of TNIK in the temporal cortex from TLE patients (*n* = 20) and control individuals (*n* = 10). TNIK was significantly down-regulated in TLE patients compared with controls (control group, 0.74 ± 0.05; TLE, 0.45 ± 0.04; *P* < 0.001) (Fig. 2A). GAPDH was used as an internal control. Immunofluorescence revealed that the TNIK protein was mainly found in neurons in the temporal cortex of TLE patients (Fig. 2B). IHC demonstrated that TNIK was mainly localized in the cytoplasm and membranes of neurons in both groups (Fig. S1A). Moreover, weaker staining was observed in the epilepsy group than in the control group (Fig. S1A).

### TNIK expression in the hippocampus and cortex of a pilocarpine-induced chronic epilepsy rat model

**Figure 3:**
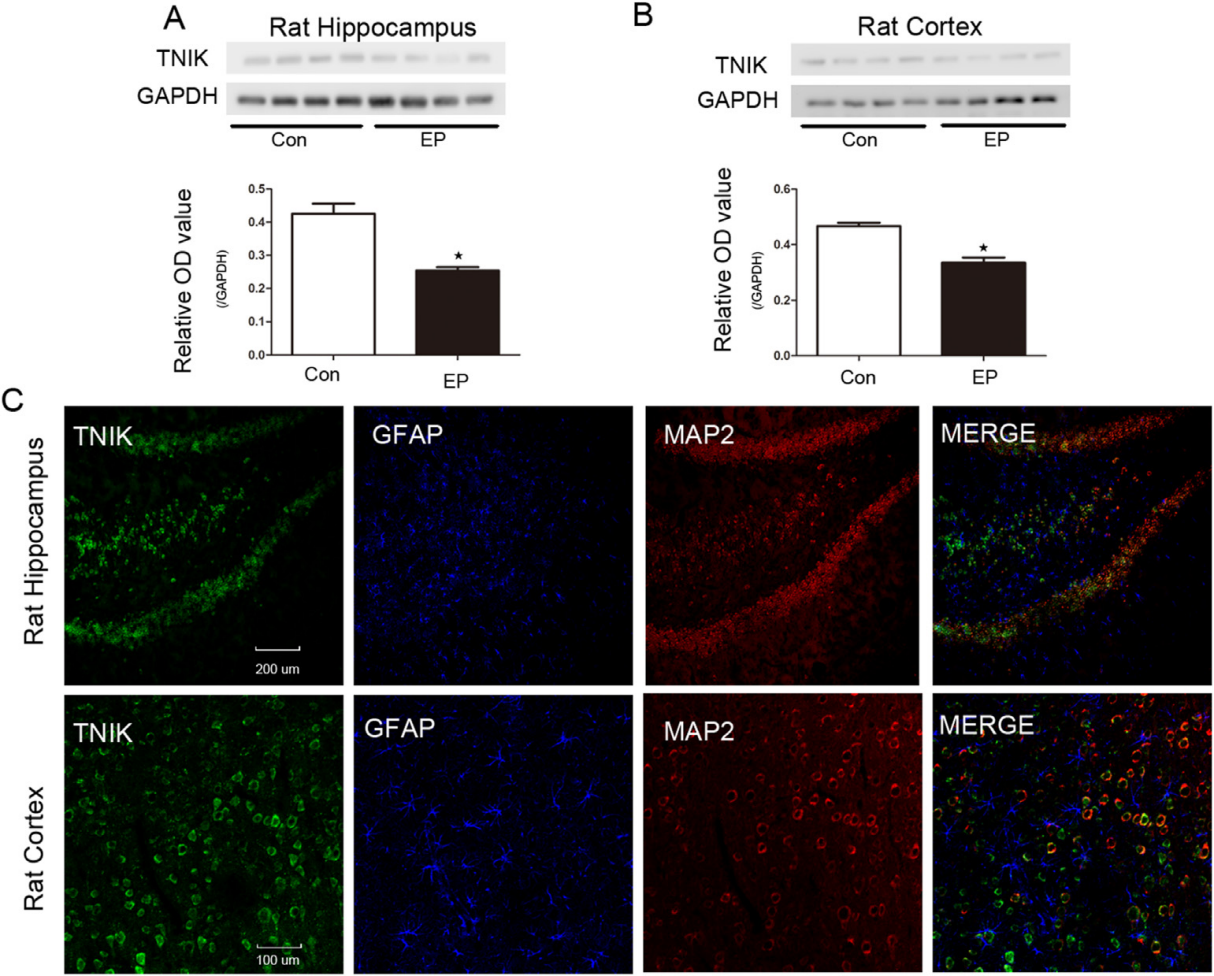
TNIK expression in the hippocampus and cortex of the pilocarpine-induced rat epilepsy model. **(A)** TNIK expression was significantly decreased in the hippocampus of the pilocarpine-induced rat epilepsy model compared with that of the control group (*n* = 6, *P* < 0.05). The relative OD ratio represents the OD ratio of TNIK relative to GAPDH. **(B)** TNIK expression was significantly decreased in the cortex of the pilocarpine-induced rat model group compared with that in the control group (*n* = 6, *P* < 0.05). The relative OD ratio represents the OD ratio of TNIK relative to GAPDH. **(C)** Triple-label immunofluorescence demonstrated that TNIK (green) and GFAP (blue) were not co-expressed in astrocytes, but TNIK (green) and MAP2 (red) were co-expressed in rat neurons (*n* = 5).

Western blotting and immunofluorescence were also used to detect the expression of TNIK in the hippocampus and cortex of a pilocarpine-induced chronic epilepsy rat model. The expression in the epilepsy rat model was similar to that found in humans. TNIK protein levels were significantly reduced in both the hippocampus (control group, 0.42 ± 0.03; epilepsy group, 0.25 ± 0.01; *P* < 0.05) and the adjacent cortex (control group, 0.46 ± 0.01; epilepsy group, 0.33 ± 0.02; *P* < 0.05) compared with the levels in the corresponding controls (control, *n* = 6; epilepsy, *n* = 6) (Fig. 3A). The OD value of TNIK was normalized to that of GAPDH. Similarly, immunofluorescence staining showed that TNIK was located in neurons rather than astrocytes (Fig. 3B). TNIK was mainly expressed in the membrane and cytoplasm of neurons. Strong staining was observed in the control group compared to the epilepsy group (Fig. S1B). In addition, we also tested the expression of TNIK in the acute phase of epilepsy in a pilocarpine-induced epilepsy rat model. The data showed that in the acute phase, there was no significant difference between the two groups (Fig. S2).

### Effect of NCB-0846 on epileptic seizures

**Figure 4:**
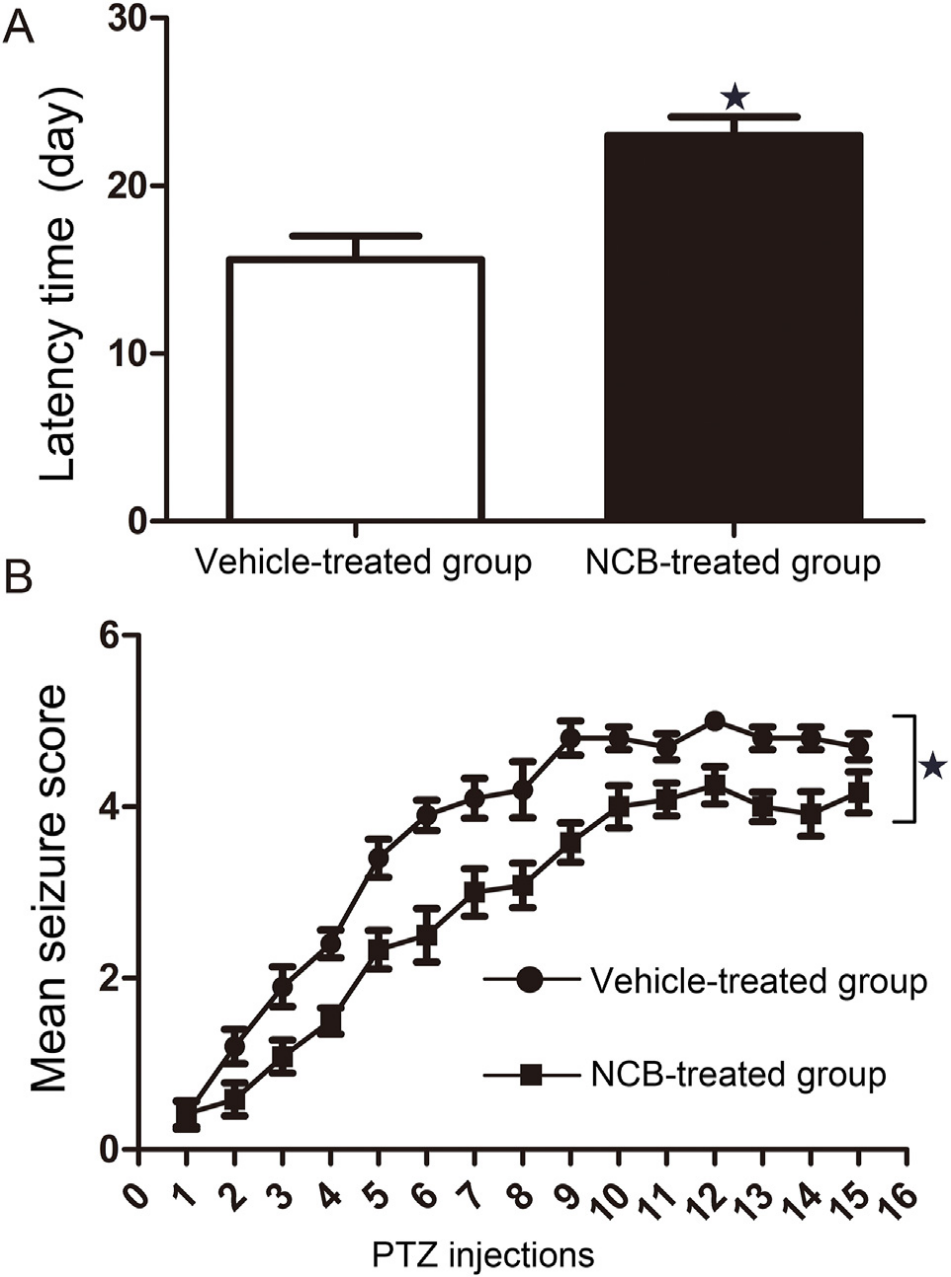
Effect of TNIK on epileptic seizures. **(A)** Behavioral data show a significantly increased latency in the NCB-0846-treated group compared with that in the vehicle-treated group (intracerebroventricularly, 10 μM, 5 μL, *n* = 10–12, *P* < 0.05). **(B)** In the PTZ kindling rat model, seizure activity was suppressed in the NCB-0846-treated group (intracerebroventricularly, 10 μM, 5 μL, *n* = 10–12, *P* < 0.05).

To assess the effect of NCB-0846 on behavioral phenotypes, a PTZ-induced epilepsy model was used. The latency time was 15.6 ± 1.4 days in the vehicle-treated group (*n* = 10) and increased to 23.0 ± 1.1 days in the NCB-0846-treated group (*n* = 12) (Fig. 4A). In addition, NCB-0846 treatment attenuated the severity of generalized tonic‒clonic seizures compared with those observed in the control group (Fig. 4B, *P* < 0.05).

### Identification of the interactome of TNIK in the rat hippocampus by LC‒MS/MS

**Figure 5:**
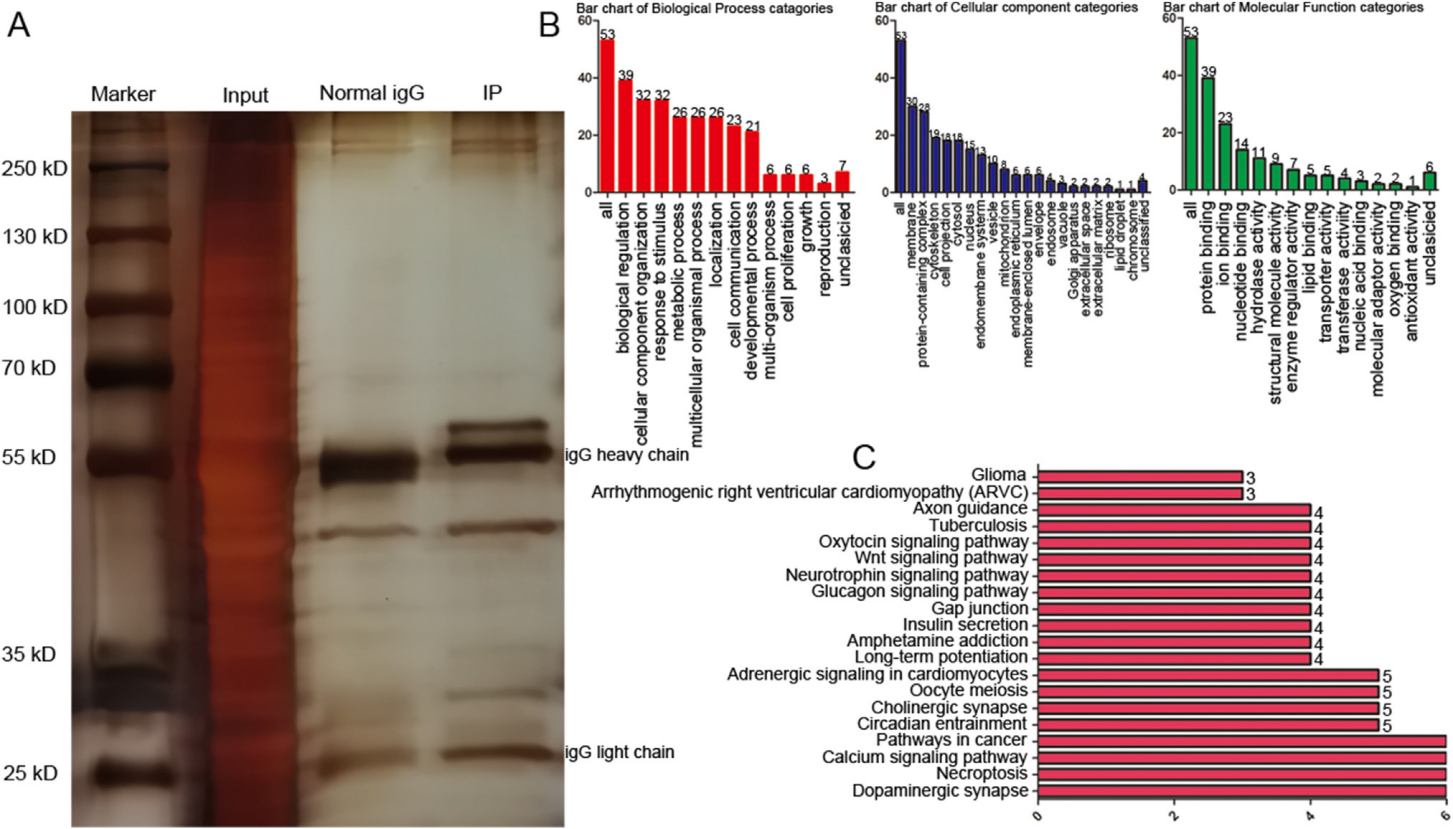
Identification, GO enrichment analysis, and KEGG analysis of TNIK-binding proteins. **(A)** Polyacrylamide gel stained with rapid silver staining. **(B)** The graph showing the enriched molecular function, cellular component, and biological process terms. **(C)** KEGG pathways of TNIK-interacting proteins.

**Figure 6:**
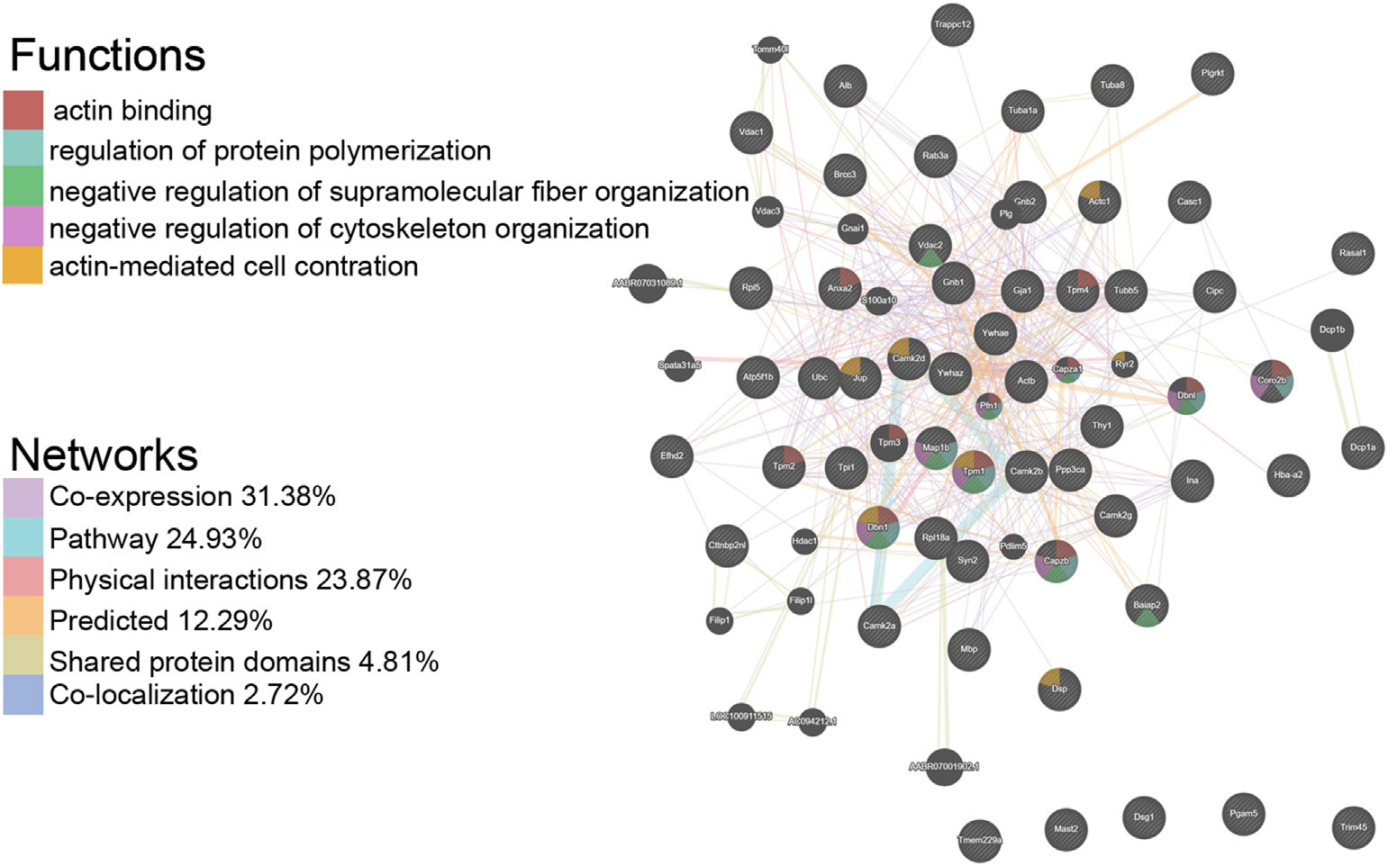
PPI networks of TNIK-interacting proteins based on GeneMANIA software. Each node represents a gene. Different connection line colors indicate the following types of protein–protein relations: coexpression, pathway, physical interaction, predicted, shared protein domains, or colocalization. Different colors of the nodes indicate the biological functions: actin binding, regulation of protein polymerization, negative regulation of supramolecular fiber organization, negative regulation of cytoskeleton organization, and actin-mediated cell contraction.

Co-IP/MS was conducted to characterize the interactome of TNIK in the rat hippocampus. Co-IP was conducted as described previously. TNIK-interacting proteins were efficiently screened from rat hippocampal lysates via SDS‒PAGE (Fig. 5A). Several bands appeared only in the Co-IP of TNIK and were not present with the control IgG. The relevant parts of the gels were cut out and saved for later MS. Sixty-three immunoprecipitated proteins were successfully identified in Mascot (shown in Table 3), including CaMKII and SYN2. The resulting gene list (in Table 3) contained 55 user IDs. Of these 55 user IDs, 53 were unambiguously mapped to the 53 unique Entrez Gene IDs. As shown in Figure 5B, the Gene Ontology (GO) slim summary was based on the 53 unique Entrez Gene IDs and analyzed for cellular component (CC), molecular function (MF), and biological process (BP) annotations. Among the 53 unique Entrez Gene IDs, 43 were annotated to the selected functional categories and used for the enrichment analysis. The reference list was mapped to 24572 Entrez Gene IDs, and 7395 IDs were annotated to the selected functional categories that were used as the reference for the enrichment analysis. Enrichment analysis in the GO domain “Biological Process” identified two predominant themes: biological regulation and cellular component organization. However, “Molecular Function” identified two predominant themes: protein binding and ion binding (Fig. 5B). KEGG pathway enrichment indicated that the identified proteins were mainly involved in the categories dopaminergic synapse, necroptosis, calcium signaling pathway, pathways in cancer, Wnt signaling pathway, long-term potentiation, neurotrophin signaling pathway, axon guidance, *etc* (Fig. 5C). Potential interactions between these identified genes were displayed using GeneMANIA analysis (Fig. 6). There were six types of interrelationships: coexpression (31.38%), pathway (24.93%), physical interactions (23.87%), predicted (12.29%), shared protein domains (4.81%), and colocalization (2.72%). The predicted functions based on a large database of functional interaction networks from multiple organisms are also listed. The top 5 predicted functions in TNIK-related genes are listed: actin binding, regulation of protein polymerization, negative regulation of supramolecular fiber organization, negative regulation of cytoskeleton organization, and actin-mediated cell contraction (Fig. 6). However, there are also several predicted functions that are important for epilepsy: positive regulation of dendritic spine development, regulation of synapse structure or activity, positive regulation of dendrite morphogenesis, and postsynaptic cytoskeleton organization (false discovery rate <0.01).

**Table 3:** Proteins identified to interact with TNIK as determined by Co-IP/MS.

Number	Protein ID	Protein name	Gene ID	Gene name
1	G3V9G3	Calcium/calmodulin-dependent protein kinase	24245	Camk2b
2	F1LMV6	Desmoplakin	306871	Dsp
3	Q4FZY0	EF-hand domain-containing protein D2	298609	Efhd2
4	F1LMV9	Coronin	300768	Coro2b
5	A0A0G2K3K2	Actin, cytoplasmic 1		Actb
6	Q91XN7	Tropomyosin alpha isoform	24851	Tpm1
7	Q6GMN2	Brain-specific angiogenesis inhibitor 1-associated protein 2	117542	Baiap2
8	P68035	Actin, alpha cardiac muscle 1	29275	Actc1
9	Q5XI32	F-actin-capping protein subunit beta	298584	Capzb
10	P11275	Calcium/calmodulin-dependent protein kinase type II subunit alpha	25400	Camk2a
11	P11730	Calcium/calmodulin-dependent protein kinase type II subunit gamma	171140	Camk2g
12	P48500	Triosephosphate isomerase	24849	Tpi1
13	P15791	Calcium/calmodulin-dependent protein kinase type II subunit delta	24246	Camk2d
14	P62260	14-3-3 protein epsilon	29753	Ywhae
15	G3V8Q2	Alpha-internexin	24503	Ina
16	Q71DI1	Dermcidin		
17	P54311	Guanine nucleotide-binding protein G(I)/G(S)/G(T) subunit beta-1	24400	Gnb1
18	Q45QL61	Guanine nucleotide binding protein beta 2		Gnb2
19	P68370	Tubulin alpha-1A chain	64158	Tuba1a
20	P09495	Tropomyosin alpha-4 chain	24852	Tpm4
21	P63329	Serine/threonine-protein phosphatase 2B catalytic subunit alpha isoform	24674	Ppp3ca
22	Q5FVG5	Similar to tropomyosin 1, embryonic fibroblast-rat, isoform CRA_c	500450	Tpm2
23	A0A0G2JSH5	Albumin	24186	Alb
24	Q6AY56	Tubulin alpha-8 chain	500377	Tuba8
25	P69897	Tubulin beta-5 chain	29214	Tubb5
26	Q6P0K8	Junction plakoglobin	81679	Jup
27	B2RZB2	Uncharacterized protein		
28	M0RAV0	Ig-like domain-containing protein		
29	A0A0G2JV65	14-3-3 protein zeta/delta		Ywhaz
30	A0A0G2K5X3	Ig-like domain-containing protein		
31	D4AE80	mRNA-decapping enzyme 1A	361109	Dcp1a
32	D3ZQ45	Desmoglein 1		Dsg1
33	P01830	Thy-1 membrane glycoprotein	24832	Thy1
34	P08050	Gap junction alpha-1 protein	24392	Gja1
35	P09895	60S ribosomal protein L5	81763	Rpl5
36	P62718	60S ribosomal protein L18a	290641	Rpl18a
37	P10719	ATP synthase subunit beta, mitochondrial	171374	Atp5f1b
38	P15205	Microtubule-associated protein 1B	29456	Map1b
39	P63012	Ras-related protein Rab-3A	25531	Rab3a
40	P81155	Voltage-dependent anion-selective channel protein 2	83531	Vdac2
41	P62804	Histone H4	29115229527736472364627	H4c2Hist1h4mH4f16
42	Q07936	Annexin A2	56611	Anxa2
43	Q9Z2L0	Voltage-dependent anion-selective channel protein 1	83529	Vdac1
44	Q07266	Drebrin	81653	Dbn1
45	Q63537	Synapsin-2	29179	Syn2
46	A0A0G2JSV6	Globin c2	360504	Hba-a2
47	B1H288	Cilia and flagella associated protein 94	297720	Casc1
48	D3ZAR2	Non-specific serine/threonine protein kinase	313819	Mast2
49	F1M269	Gp_dh_N domain-containing protein		
50	D3ZHY9	RAS protein activator like 1 (GAP1 like) (predicted)	360814	Rasal1
51	I7FKL4	Myelin basic protein	24547	Mbp
52	M0RDP3	Uncharacterized protein		
53	Q63654	Polyubiquitin		UBC
54	G3V9N1	RCG21137		Pgam5
55	Q699Y1	60 kDa chaperonin		
56	D4ACS9	RCG27978	685756	Tmem229a
57	Q6S398	Plectin 8		
58	D3ZE49	Trafficking protein particle complex 12	314013	Trappc12
59	D4A8X8	CTTNBP2 N-terminal like (predicted), isoform CRA_a	310760	Cttnbp2nl
60	D4ACN8	Plasminogen receptor (KT)	293888	Plgrkt
61	D4A7S9	Tripartite motif protein 45 (predicted), isoform CRA_b	295323	Trim45
62	B2RYM5	Lys-63-specific deubiquitinase BRCC36	316794	Brcc3
63	D4A489	CLOCK-interacting pacemaker	314330	Cipc

### Validation of selected TNIK protein–protein interactions (PPIs) by Co-IP followed by Western blot

**Figure 7:**
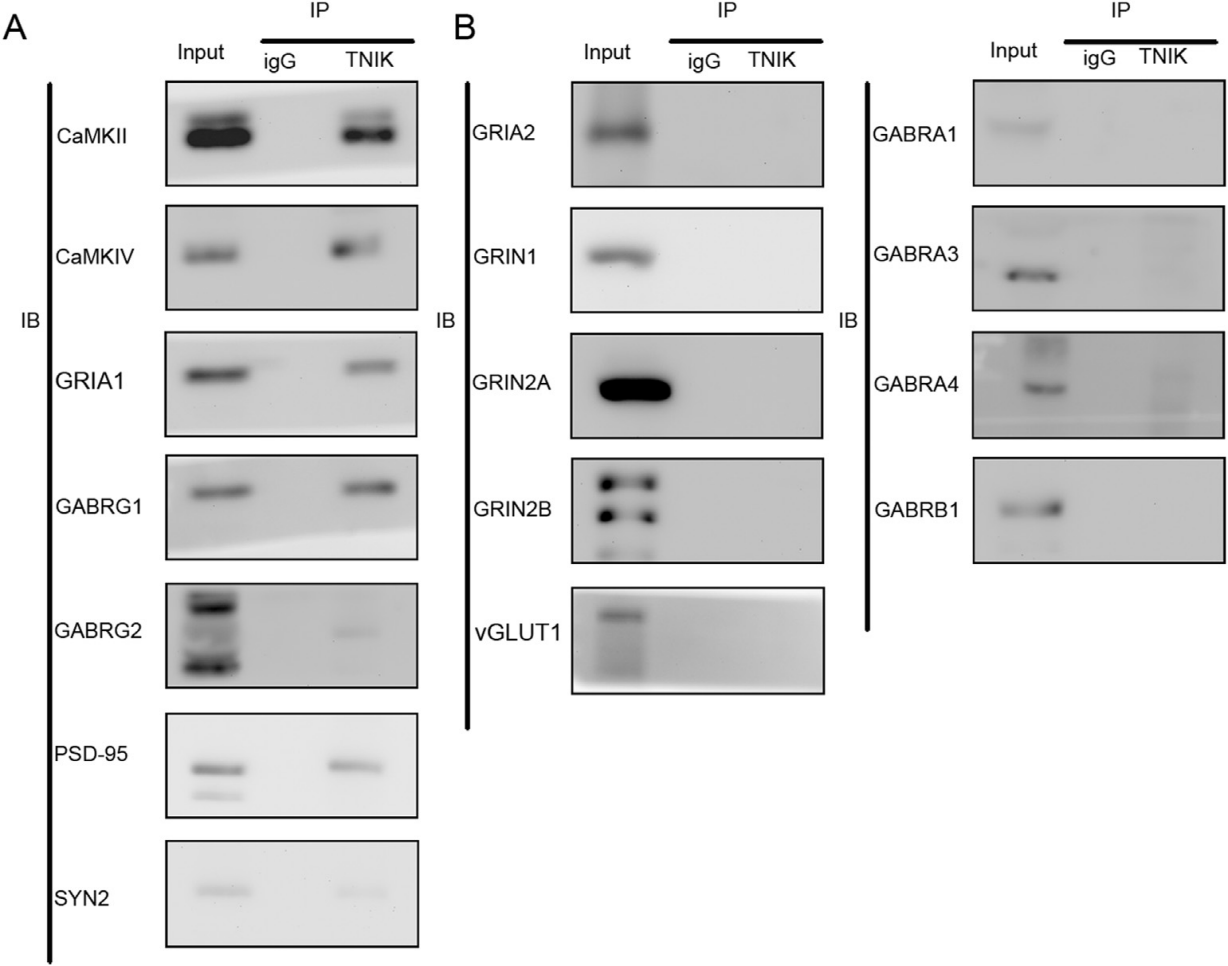
Verification of interaction via Western blot analysis. Co-IP was conducted with anti-TNIK antibody, and mouse IgG was used as a negative control. Blots were probed with the respective antibody. Immunoprecipitation, IP; immunoblot, IB.

Data from Co-IP/MS showed that TNIK is associated with CaMKII and SYN2. Western blotting was used to verify the association between CaMKII, SYN2, and TNIK. The data showed that CaMKII and SYN2 interact with TNIK (Fig. 7A). In addition, CaMKIV was also correlated with TNIK. A previous study also showed that TNIK was associated with glutamic receptors. Therefore, in this study, the correlations of TNIK with the main subunits of NMDAR, AMPAR, and GABA receptor (GABAR) were also tested by western blotting. Western blotting data showed that TNIK was associated with GIRA1, as well as with GABRG1, GABRG2, and PSD-95 (Fig. 7A). The data showed that TNIK was not associated with GRIA2, vGlut1, GRIN1, GRIN2A, GRIN2B, GABRA1, GABRA3, GABRA4, or GABRB1 (Fig. 7B). However, the other potential interactors in Table 3 should be verified in the future.

### Effect of TNIK on the expression of GIRA1, pGIRA1 (S845), CaMKII, CaMKIV, GABRG1, and GABRG2

**Figure 8:**
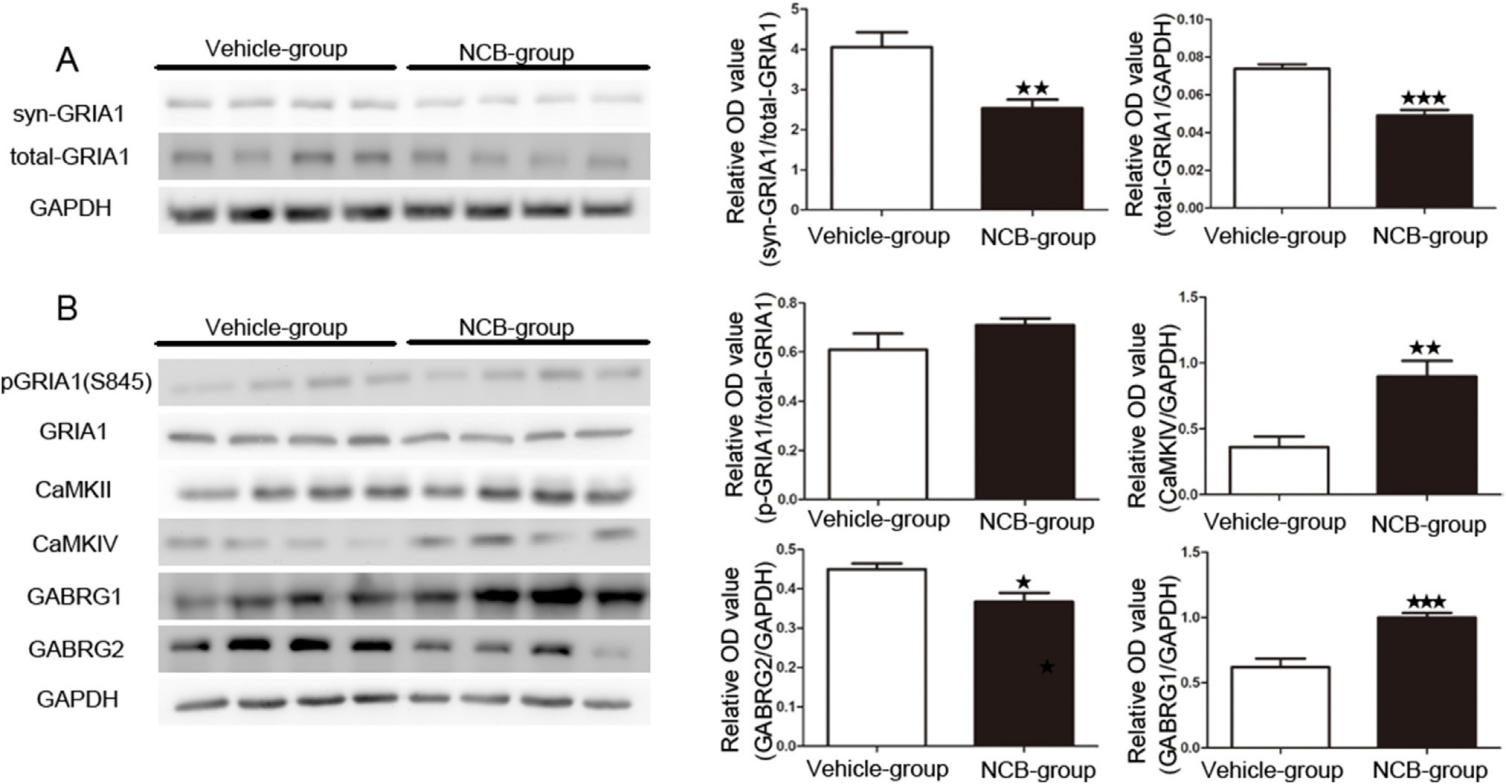
Effect of NCB-0846 on the redistribution of GRIA1. **(A)** The ratio of synaptic/total GRIA1 decreased significantly in the NCB-0846-treated group compared with the vehicle-treated group (*n* = 6, *P* < 0.01). The expression of total GRIA1 decreased significantly in the NCB-0846-treated group (*n* = 6, *P* < 0.001). **(B)** The ratio of pGRIA1 (S845) to total GRIA1 in whole-cell lysate was unchanged between the two groups (*n* = 6, *P* > 0.05). The expression of total CaMKIV and GABRG1 increased significantly in the NCB-0846-treated group (*n* = 6, *P* < 0.01, *P* < 0.001, respectively). The expression of total GABRG2 decreased significantly compared with that in the control group (*n* = 6, *P* < 0.05).

Excitatory postsynaptic transmission is mainly regulated by NMDARs and AMPARs. Inhibitory synaptic transmission is mainly regulated by GABAR. To investigate the potential mechanism by which TNIK influences animal phenotypes, animal hippocampi were collected after NCB-0846 treatment (intracerebroventricularly) for 7 consecutive days in naïve rats. The subcellular and total expression levels of GRIA1 were tested and are shown in Figure 8A. The data showed that a significant reduction in GRIA1 in the total lysate and PSD was observed in the NCB-0846 treatment group compared with the control group (total lysate: vehicle group, 0.073 ± 0.002; NCB-0846 treatment group, 0.049 ± 0.002, *P* < 0.001; PSD: vehicle group, 4.05 ± 0.366; NCB-0846 treatment group, 2.52 ± 0.217, *P* < 0.01; *n* = 6; Fig. 8A). Then, the ratio of pGRIA1 (S845) to total GRIA1 was tested in the total lysate. The data showed that there was no significant difference in this ratio between these two groups (vehicle group, 0.609 ± 0.065; NCB-0846 treatment group, 0.71 ± 0.025; *n* = 6, *P* > 0.05; Fig. 8B). In this study, CaMKII, CaMKIV, GABRG1, and GABRG2 were found to interact with TNIK. We also tested the effect of NCB-0846 on their expression in hippocampal total lysates. The data showed that CaMKIV, but not CaMKII, increased significantly in the NCB-0846-treated group (vehicle group, 0.361 ± 0.08; NCB-0846 treatment group, 0.898 ± 0.11, *P* < 0.01; *n* = 6; Fig. 8B). GABRG2 decreased significantly in the NCB-0846-treated group (vehicle group, 0.449 ± 0.015; NCB-0846 treatment group, 0.367 ± 0.022, *P* < 0.05; *n* = 6; Fig. 8B), while GABRG1 increased (vehicle group, 0.617 ± 0.065; NCB-0846 treatment group, 0.99 ± 0.034, *P* < 0.001; *n* = 6; Fig. 8B).

## Discussion

To our knowledge, this study provides the first evidence that TNIK participates in the pathology of epilepsy and that NCB-0846 plays a critical role in epileptic seizures. In particular, we found that (i) TNIK expression decreased significantly in TLE patients and epileptic rats; (ii) TNIK was primarily located in the neurons; (iii) the TNIK inhibitor NCB-0846 exerted anticonvulsant effects; (iv) Co-IP/MS identified 63 proteins that might interact with TNIK in the rat hippocampus, of which CaMKII was prominent; (v) Co-IP studies confirmed that TNIK could interact with CaMKII, SYN2, CaMKIV, GRIA1, as well as GABRG1, GABRG2, and PSD-95; (vi) TNIK inhibition decreased GRIA1 in total lysis and PSD, which might contribute to the delay in the PTZ kindling process; (vii) The pGRIA1 (S845)/GRIA1 ratio was unchanged after NCB-0846 treatment; and (viii) NCB-0846 treatment increased the total CaMKIV and GABRG1 but decreased GABRG2.

TLE is the most common and devastating form of intractable epilepsy that is resistant to commercial AEDs. Therefore, the identification of effective small molecules or therapeutic targets has become a focus of epilepsy research. Although multiple mechanisms are involved in the development of epilepsy, the imbalance of E/I in the central nervous system (CNS) is a common pathophysiological change in epilepsy. Recent studies have shown that TNIK binds with NMDAR and DISC1, which play important roles in schizophrenia. These findings led us to hypothesize that TNIK dysfunction might be involved in the pathology of epilepsy.

First identified in 1999, TNIK is highly expressed in PSD and synaptosomal fractions, which supports its role in glutamatergic signaling.^10^ TNIK loss of function causes learning defects and alters synaptic function.^8^ A number of circumstantial lines of evidence imply that TNIK is involved in schizophrenia and bipolar disorder. Data from the Human Protein Atlas show that TNIK is expressed extensively in the human body, especially in the brain (https://www.proteinatlas.org/ENSG00000154310-TNIK/tissue). TNIK has been identified as highly expressed in the mammalian cortex and hippocampus, as confirmed by the Allen Brain Atlas (http://mouse.brain-map.org/gene/show/154334). TNIK mRNA was shown to be up-regulated in the dorsolateral prefrontal cortex of schizophrenia patients^41^ and in lymphoblastoid cell lines from bipolar disorder patients when compared with their healthy monozygotic twins.^30^ This expression pattern and previous data might imply TNIK's important role in epilepsy. To test our hypothesis, we first tested TNIK protein expression in brain samples from TLE patients and found that TNIK protein was truly expressed in the human brain. Moreover, TNIK expression is significantly down-regulated in TLE patients, which provides evidence that TNIK might be involved in epilepsy. In view of the design limitation imposed by the control group, which was selected from patients needing surgery for intracranial pressure, we sought to confirm the results by measuring the TNIK expression in the cortex of a pilocarpine-induced chronic rat epilepsy model, which shows specific changes in the selectively vulnerable hippocampal formation. Surprisingly, TNIK expression was also significantly down-regulated in the cortex of the epileptic rat model, which is consistent with the findings in TLE patients. However, the hippocampus is considered the main region of concern in epilepsy. Then, we tested and found that TNIK protein expression was down-regulated in the hippocampus of the epileptic rat model. Moreover, our immunohistochemical data showed that TNIK is weaker in samples with epilepsy than in the controls, whether in patients or in rats (Fig. S1). Furthermore, the data from immunofluorescence showed that TNIK colocalized with MAP2, a marker for neurons, but not with GFAP, a marker for astrocytes. This is consistent with a previous study showing that TNIK is predominantly expressed in the brain in many regions and that TNIK is a neuronal protein.^15^ Together, these data showed that TNIK might be an important factor involved in epilepsy.

To test the potential effect of TNIK on epilepsy, a chronic PTZ-kindled epilepsy rat model was chosen. Chronic PTZ-kindled epilepsy models represent chronic models in which repeated subconvulsive doses (30–35 mg/kg) of PTZ lead to the intensification of seizure activity and enhanced seizure susceptibility similar to that in human epilepsy.^42^ PTZ-kindled chronic epilepsy models have been extensively used to assess the protective effects of numerous AEDs when administered before the start of the kindling procedure. The other reason for this choice is that PTZ's proconvulsant function was credited to its effect on stimulating seizure activity by blocking GABA-mediated transmission in the CNS,^42^ and administration at a subconvulsive dose in adult rodents was found to induce TLE.^43^ In addition, a previous paper showed that PTZ chronically administered at a subconvulsive dose is typically used to identify the progression of epileptogenesis.^44^ We found that inhibition of TNIK alone did not cause epileptic seizures or epileptic discharges. The TNIK inhibitor NCB-0846 prolonged the latency time and decreased the severity of seizure activity. These data suggest that down-regulation of TNIK decreased seizure susceptibility and delayed the process of epileptogenesis.

Bioinformatics data (https://thebiogrid.org/576981/summary/mus-musculus/tnik. html) shows that TNIK in mice may be correlated with GRIA1, GRIA2, GRIN1, GRIN2A, GRIN2B, and PSD-95,^45^^,^^46^ which play important roles in epilepsy; in rats, however, the only correlate of TNIK is GRIA1 (https://thebiogrid.org/254828/table/rattus-norvegicus/tnik.html). MS-based proteomics has emerged as a great way to define global changes in protein abundance.^47^ Combined with affinity enrichment strategies, MS-based proteomics can identify interacting partners of key proteins linked to disease pathogenesis.^48^ To better understand the mechanisms underlying the function of TNIK in rat hippocampal neurons, we searched for TNIK-interacting proteins by Co-IP/MS. Bound proteins were eluted, separated by SDS/PAGE, and subjected to MS analysis. In this study, Co-IP/MS identified 63 TNIK-associated proteins. The “Molecular Function” subset of the GO slim identified two predominant themes: protein binding and ion binding, which is consistent with TNIK also containing scaffolding domains. KEGG pathway enrichment indicated that TNIK-related proteins were mainly involved in dopaminergic synapses, the Wnt signaling pathway, axon guidance, the calcium signaling pathway, and long-term potentiation, all of which are important in epilepsy. Consistently, TNIK has already been implicated in cell proliferation, cytoskeleton organization, neuronal dendrite extension, and glutamate receptor regulation *in vitro*.^7^^,^^11^^,^^12^ In addition, KEGG pathway enrichment indicated that TNIK-related proteins were also involved in cancer pathways, which is well verified in previous data.^17^, ^18^, ^19^, ^20^, ^21^, ^22^, ^23^, ^24^, ^25^ GeneMANIA is an online analysis tool for deriving hypotheses based on gene functions.^40^ GeneMANIA is a flexible user-friendly website for PPI network construction based on genomic, proteomic, and gene function data. GeneMANIA can illustrate the potential relationship among a list of genes by constructing an interactive network. We also conducted a PPI network analysis among the identified genes and explored their interactions. The identified genes showed strong correlations with actin binding, cytoskeleton organization, and dendritic spine morphogenesis, which are important for epilepsy.

According to the data from Co-IP/MS, we conducted IP followed by western blotting to further validate the key candidate interacting molecule. This effort yielded a protein known as CaMKII, which is a highly abundant serine/threonine kinase comprising a signiﬁcant fraction of total protein in the mammalian forebrain and forming a major component of the PSD^49^ and is essential for synaptic plasticity and memory consolidation. A previous study showed that CaMKII, located in excitatory synapses, is involved in the etiology of seizure activity. In addition, CaMKII activity (autophosphorylation of CaMKII) decreased following the onset of epileptiform activity in a variety of models.^50^^,^^51^ Evidence shows that loss of CaMKII activity at synapses is linked to the onset of epileptogenesis.^52^ However, some researchers have shown that total protein levels of CaMKII were not decreased following the onset of recurrent seizure activity.^53^^,^^54^ Some researchers have even shown an increase in CaMKIIα total protein at prolonged time points following seizure onset.^49^ CaMKII is essential for NMDAR-dependent hippocampal long-term potentiation,^55^ long-term depression, and the function of inhibitory synapses.^56^ In addition to its signaling and enzymatic function, CaMKII also plays a structural role via direct interaction with actin filaments, which is one of the main results of our GO enrichment analysis. Although not detected in our Co-IP/MS data, CaMKIV, another Ca^2+^/CaM-binding protein that plays pivotal roles in neurons, was detected to be potentially associated with TNIK. To our surprise, CaMKIV was also correlated with TNIK in the rat hippocampus. One reason might be that CaMKII and CaMKIV have quite similar substrate specificity determinants. The other is that TNIK is an essential activator of Wnt target genes,^17^ while a previous study showed that CaMKIV is a target gene of the Wnt signaling pathway.^57^ After NCB-0846 treatment, the expression of CaMKIV increased significantly in hippocampal total lysates. Previous data showed that activation of CaMKIV could induce a reduction in AMPA receptor subunits in cultured cells.^58^ Our data also showed that PSD-95 interacts with TNIK, which could indicate that TNIK could regulate PSD-95 ^9^.

The imbalance of E/I neurotransmitter function in the CNS of patients with seizures is the main biochemical and biophysical hallmark of epilepsy. A previous paper showed that this E/I imbalance is a result of haploinsufficiency of TNIK.^8^ Evidence has shown that TNIK is located in the PSD of synapses.^13^, ^14^, ^15^ Previous data and our data showed that TNIK is associated with GRIA1. GRIA1 contains phosphorylation sites for CaMKII, which regulate its synaptic insertion.^59^ Then, in this study, synaptic GRIA1 was detected in the subcellular fraction, and the data showed that GRIA1 was down-regulated in total lysates and PSD. In this study, biochemical data showed that GRIA1 prevented trafficking to the PSD after NCB-0846 treatment. This aberrant expression and trafficking of GRIA1-containing receptors may be attributable to the overexpression of CaMKIV. The ratio of pGRIA1/GRIA1 was not changed significantly. Although speculative, TNIK modulates synaptosomal glutamatergic receptors through scaffolding domains but not enzymatic activity. Synapsins play an important role in the clustering of synaptic vesicles (SVs), and their perturbations lead to disruption of the organization of SV pools and to an increase in synaptic depression.^60^ SYN2, as a synapsin, can contribute to the pathophysiology of epilepsy based on basic work^61^ and clinical findings.^62^ In our study, MS and Western blot data showed that SYN2 might interact with TNIK, further explaining the potential mechanism of TNIK involvement in epileptic phenotypes.

The abnormal structure of hippocampal fractions, even including the content of synaptosomes, disturbed the balance of excitability/inhibition in the CNS and caused refractory spontaneous epileptic activity. Synaptic NMDAR, AMPAR, and GABARs with various subunit compositions are the main receptors mediating synaptic transmission and are not static but change dynamically in response to neuronal activity, which can contribute to neuropsychiatric disorders if this activity is disturbed. Synaptic inhibition, mediated by GABA receptors, is vital for the efficient control of network excitability and E/I balance, which is important for normal brain function and epilepsy. Inhibitory synapses require the stabilization of postsynaptic GABARs, and their modulation can be achieved by the clustering of GABARs.^63^ To investigate the potential role of TNIK in postsynaptic assembly, we examined the main subunits of GABARs that directly or indirectly interact with TNIK using Co-IP. The data showed that TNIK interacted with GABRG1 and GABRG2. In addition, the expression of GABRG1, but not GABRG2, was up-regulated in total hippocampal lysates after NCB-0846 treatment. Previous studies have shown TNIK enrichment at excitatory synapses.^13^, ^14^, ^15^ Little is known regarding their localization to inhibitory synapses. Our data showed that TNIK was associated with the main subunits of GABARs. Although a previous study showed that mIPSCs were normal in TNIK knockout mice,^8^ the participation of TNIK in inhibitory synapses should be illuminated in the future.

Although our results thus far indicate the participation of TNIK activity in seizure behavioral and molecular abnormalities, the mechanism of constitutive TNIK signaling in epilepsy should be investigated further in the future using genetic knockout or overexpression of TNIK. Whether TNIK directly binds to CaMKII, CaMKIV, GRIA1, and GABARs should be investigated in the future. Further analyses of interacting proteins should aid in elucidating the biological functions of TNIK in the progression of epilepsy. TNIK down-regulation might be an endogenous protection mechanism for TLE patients, and its agonists or overexpression might be deleterious to epilepsy patients. Nevertheless, in this study, TNIK biology facilitated our understanding of the submolecular dysfunction underlying epilepsy.

## Author contributions

ZFD, XT, and YKZ conceived the project and designed the experiments. MW, YXG, QBL, BZF, HZ, XT, and YKZ performed the experiments. MW, YXG, XKL, and QXK analyzed the data. MW, XT, and YKZ wrote the manuscript. All authors revised and approved the final version of the manuscript.

## Conflict of interests

The authors declare that the research was conducted in the absence of any commercial or financial relationships that could be construed as a potential conflict of interest.

## Funding

This work was supported by grants from the 10.13039/501100001809National Natural Science Foundation of China (No. 81901324, 82001378, 82071395), 10.13039/501100002858China Postdoctoral Science Foundation (No. 2021M693246), joint project of Chongqing Health Commission and Science and Technology Bureau (Chongqing, China) (No. 2023QNXM009), Science and Technology Research Program of Chongqing Education Commission of China (No. KJQN202200435), Chongqing Talents: Exceptional Young Talents Project (Chongqing, China) (No. CQYC202005014), 10.13039/501100005230Natural Science Foundation of Chongqing, China (No. cstc2021ycjh-bgzxm0035, CSTB2022NSCQ-LZX0038), and Key Research and Development Projects of Jining City, Shandong, China (No. 2021YXNS057).

## Data availability

The original contributions presented in the study are included in the article or supplementary material, and further inquiries can be directed to the corresponding author.

## Figures and Tables

**Figure:**
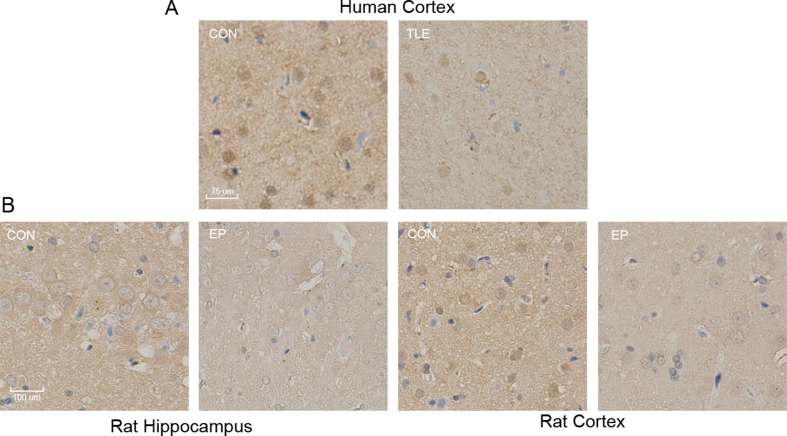
**Figure S1** IHC staining of TNIK in rats and patients. (A) IHC staining of TNIK was observed in the cortex and hippocampus in both groups, but weaker staining was observed in rats with epilepsy. (B) IHC staining of TNIK was observed in the cortex in both groups, but weaker staining was observed in TLE patients.

**Figure:**
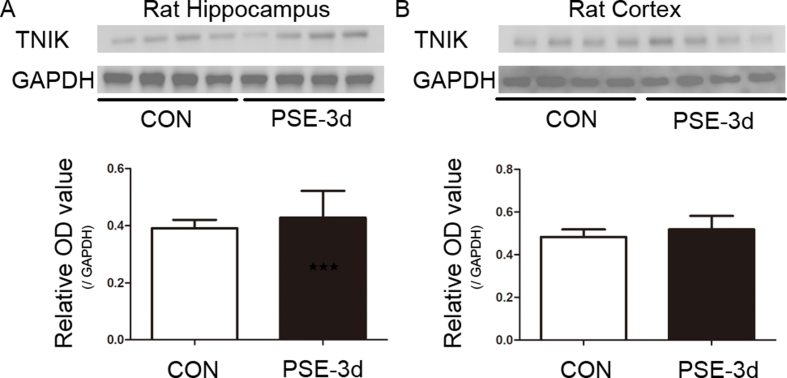
**Figure S2** TNIK expression in the hippocampus of the pilocarpine-induced rat epilepsy model (acute phase: PSE-3d). There was no significant difference between the two groups in the acute phase of epilepsy (PSE-3d: 3 days post status epilepticus) (n = 6, *P* > 0.05). The relative OD ratio represents the OD ratio of TNIK relative to GAPDH.
